# Evaluation of MicroRNA-99a and MicroRNA-205 Expression Levels in Bladder Cancer

**DOI:** 10.22088/acadpub.BUMS.6.2.3

**Published:** 2017-07-08

**Authors:** Sajjad Mohammad Ganji, Massoud Saidijam, Razieh Amini, Seyed Habibollah Mousavi-Bahar, Nooshin Shabab, Saman Seyedabadi, Ali Mahdavinezhad

**Affiliations:** 1 *Research Center for Molecular Medicine, Department of Genetics and Molecular Medicine, Hamadan University of Medical Sciences, Hamadan, Iran.*; 2 *Urology and Nephrology Research Center, Shahid Beheshti Hospital, Hamadan University of Medical Sciences, Hamadan, Iran.*

**Keywords:** microRNAs, neoplasms, urinary bladder

## Abstract

Bladder cancer is the second most common cancer in the genitourinary tract, showing often recurrence and progress into invasive states. Epigenetic changes, such as microRNA alteration are involved in bladder cancer tumorigenesis through a variety of signaling pathways. The epigenetic state depends on geographic and lifestyle conditions. The aim of this study was to investigate the expression level of microRNA-99a and microRNA-205 in bladder cancer in Iranian populations and to determine the relationship between their expressions with clinicophatological features**. **36 patients with bladder cancer were included in the study. The control group was the healthy adjacent tissue of the same patients. Total RNA was extracted from approximately 50 mg tissue using TRIzol reagent. cDNA was synthesized and Real-Time PCR was carried out using specific primers. The Unisp6 rRNA was used as a reference gene. A significant decrease was found in the expression level of miR-99a in tumor samples, compared to healthy adjacent tissues (P<0.001). The increased expression level of miR-99a was significantly associated with muscle invasion (P=0.02). The receiver operating characteristic (ROC) analysis for miR-99a showed AUC value equal to 0.944, with specificity of 97%, sensitivity of 91%, and cut off value of 8.31 (P<0.001). A significant association was found between smoking and miR-99a (P=0.04) and miR-205 (P= 0.01) expression levels. Dramatic down-regulation of miR-99a in bladder cancer tissues confirmed the tumor suppressor role of miR-99a in bladder cancer. A higher amount of miR-99a expression was associated with invasive bladder cancer. According to ROC analysis, miR-99a could be considered as a valuable diagnostic biomarker.

Bladder cancer (BC) is the most common malignancy of the urinary tract and the fourth most common cancer in developed countries (1). Smoking is one of the most well-known risk factors for BC. Smoking causes 31% and 14% of BC-related deaths in men and women around the world, respectively (2). It was estimated that 2.4% of the men and women face BC diagnosis throughout their lives (3). In spite of the high incidence and prevalence, treatment of bladder cancer has not changed in the nearly recent three decades (4). Cystoscopy and transurethral resection, considered as invasive processes, are generally applied for diagnosis, treatment and follow-up of patients (5). By 2025, up to 45% increase of cancer incidence is expected in developing countries (6). Currently, there is no standard screening test to identify individuals at risk of BC (2). Carcinogenesis and progression of BC are caused by multiple genetic and epigenetic changes (7). Given the foregoing, the accurate mechanism of bladder carcinogenesis has not clearly been determined to date. Therefore, there is a need for understanding the process of genetic and epigenetic alterations to identify molecular biomarkers for diagnosis, prognosis and therapeutic decision (8).

MicroRNAs (MiRNAs) are a group of short (approximately 18-22 nucleotides) non-coding single-stranded RNA molecules that normally serve as a negative regulator of gene expression during different biologic processes including cell differentiation, proliferation, death, metabolism, apoptosis, carcinogenesis, immune response and energy homeostasis (9-11). Some miRNAs act as tumor suppressors while others serve as oncogenes during cancer development and progression (12-14). MiRNA expression in BC was determined for the first time, by Gottardo et al. (13). MicroRNA-205 (miR-205), located at the second intron of the *LOC642587* locus in human chromosome 1(1q32.2), is frequently silenced in advanced cancer. Up-regulation of miR-205 targets, including Zinc finger E-box-Binding homeobox *(ZEB) 1*, *ZEB2*, *VEGF-A* and E-cadherin, can induce epithelial to mesenchymal transition (15, 16). MicroRNA-99a (miR-99a) belongs to the miR-99 family. Mir-99a is organized in a cluster within human chromosome 21 (21q21.1). Changes in miR-99 family expression in the lung, liver, ovarian, bladder, and prostate cancers have been reported previously; miR-99a and its targets, such as suppression of tumorigenicity 5 (*ST5*), m-TOR, fibroblast growth factor receptor 3 (*FGFR3*), and insulin-like growth factor 1, are involved in cancer-related processes, and also miR-99a serves as tumor suppressor in most cases of various cancers (17-19). Furthermore, Yougang Feng et al. indicated that miR-99a might act as a tumor suppressor, since it is under-expressed in BC development (20). However, another study has reported a higher expression level of four miRNAs (let-7 c, miR-125b, miR-193a, and miR-99a) in muscle invasive bladder cancer (MIBC) (21). The study of Zhenqiang Fang et al. indicated that plasma miR-205 was up-regulated in BC, compared with healthy controls and also in MIBC compared to nonmuscle invasive bladder cancer (NMIBC) (22). Another study conducted by X Sun et al. showed the decreased expression level of miR-205 in BC samples compared to normal samples. Besides, they reported that expression of miR-205 in cancer tissues was significantly suppressed by about 80% compared with control samples (23).

Although there is a controversy on miR-99a expression levels in cancer and normal tissues, and also differences in miR-99a expression in MIBC and NMIBC, due to the probable important role of miR-99a and miR-205 in BC pathophysiology, these miRNAs have great potential as diagnostic and prognostic biomarkers. The purpose of this study was to investigate the expression of miR-99a and miR-205 in tissue samples isolated from human BC and their healthy adjacent counterparts and their relationship with clinicopathological characteristics such as muscle invasion, tumor grade and recurrence in the west of Iran.

## Materials and methods

This case- control study was approved by the Ethics Committee of Hamadan University of Medical Sciences (Hamadan, Iran) in accordance with the last revision of Declaration of Helsinki declaration. All patients signed a written informed consent. The study population consisted of 36 newly-diagnosed untreated patients with transitional cell carcinoma, which were confirmed by pathology results. Samples were taken from patients referred to Shahid Beheshti and Bu-Ali Hospitals (Hamadan, Iran) during 2013-2014. In total, the sample size consisted of 36 tumor samples and 36 healthy adjacent tissues from the same patients (10 cm away from the tumor bed) as control samples. The isolated samples, after washing by cool and sterile normal saline, were frozen immediately in liquid nitrogen, and kept at -80 °C freezer until RNA extraction.

Inclusion and exclusion criteria as well as collection of clinical specimens were reported previously (24). Patients included 3 women and 33 men with an average age of 71 years (44-91). Most patients were 60 to 80 years old, representing 61.1% of the total population. According to pathology results, 13 patients were diagnosed with MIBC and the others were NMIBC. Other clinicopathological information including grade, smoking and recurrence are given in Table 1.

**Follow-up protocol**

All patients were followed up until August, 2016 every 3 months via urine cytology and ultrasonography. Transurethral resection was applied when abnormal cytology or suspected lesion was observed. Out of 36 patients, 4 died during follow up periods, and 16 cases showed recurrence.

**RNA quality and quantity control**

Total RNA was extracted from approximately 50 mg tissue using the Trizol reagent. RNA concentration and purity was determined by optical density measurement using a NanoDrop spectrophotometer (Bio-TeK, USA). Electroph-oresis was performed on 1% agarose gel to assess the quality of RNA samples.

**cDNA synthesis** **and quantitative Real-Time** **PCR**

One step miRNA specific cDNA synthesis was performed using 2 µg of RNA, according to the kit protocol (miRCURY LNA Universal RT microRNA PCR Universal cDNA Synthesis kit II, Exiqon Company cat no: 203301**)**.  Expression of desired genes (miR-99a and miR-205) was carried out by using miRCURY LNA Universal RT microRNA PCR ExiLENT SYBER GREEN master mix (Exiqon Company, cat no: 203403), according to the company’s instructions in a CFX96 real- time PCR detection system (Bio-Rad, USA).

All quantitative Real-time PCR reactions were run in duplicate. UniSp6 rRNA was used as a reference gene to normalize Ct values because of the non- differential expression level in tumor and healthy adjacent samples.

**Statistical analysis**

Kolmogorov-Smirnov test was used to evaluate the normal distribution of data. Because the variables were not normally distributed, the equivalent non-parametric Wilcoxon paired t-test and Mann-Whitney test were used to compare variables between cancer and normal tissues, MIBC and NMIBC, smoker and nonsmoker patients as well as among tumor grades. The 2^(-ΔΔ CT)^ method was used for relative quantification of miRNA expression. A p-value less than 0.05 was considered to be statistically significant.

**Table 1:** Clinicopathological characteristics of the patients

	**Sex**		**Smoking**		**Grade**			**Muscle invasion**		**Recurrence**		**Carcinogen exposure** ¶¶¶¶	
	Male	Female	Yes	No	PUNLMP¶	LG¶¶	HG¶¶¶	Yes	No	Yes	No	Yes	No
Number	33	3	25	11	2	17	17	13	23	16	20	10	26
Percent (%)	91.7	8.3	69.4	30.6	5.6	47.2	47.2	36.1	63.9	44.4	55.6	27.8	72.2

¶ papillary urothelial neoplasm of low malignant potential;

¶¶ low grade;

¶¶¶ high grade;

¶¶¶¶ asbestos and active chemical colors.

## Results

**Expression of miR-99a and miR-205 in tumor and healthy adjacent tissues**

**Fig. 1:**
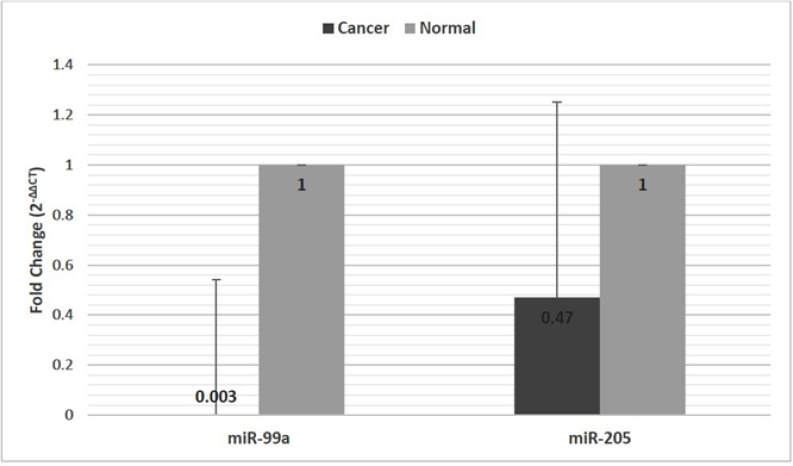
The relative expression of miR-99a and miR-205 in cancer and control tissues. The data were shown as Mean ± SE (Standard Error).

The mean Ct values of Unisp6 rRNA in case and control groups were 18.98± 1.65 and 19.22± 1.24, respectively (P=0.23). Therefore, it was a suitable choice as a reference gene to normalize gene expression between the study groups. According to the results of the study, the overall miR-99a expression level significantly decreased in tumor tissues in comparison with the normal tissues (P<0.001) (Table 2). Reduced miR-99a expression was observed in 97.2 % of the samples. The expression level of miR-99a was about 333 times lower in tumor tissues in comparison with normal tissue samples (Figure 1). MiR-205 expression decreased in 58.4% of samples while overall miR-205 expression was about 2 times lower in tumor tissues than that in normal tissue (Figure 1). Nonetheless, no statistically significant difference was observed between case and control groups regarding miR-205 expression (P=0.38) (Table 2).

**Association between muscle invasion, grading, and miRNA expression**

MiR-99a was found to be overexpressed in tumors with MIBC (P= 0.02) (Table 3). The fact that there was no association between tumor grade and miR-99a expression (P= 0.05) (Table 4), miR-99a expression was higher in high grade tumors in comparison with papillary urothelial neoplasms of low malignant potential and low grade. No relationship was observed between deregulation of miR-205 and muscle invasion (P= 0.052) (Table 3) as well as between miR-205 expression and the grade of the tumor (P= 0.87) (Table 4).

**Association between smoking, carcinogen exposure, recurrence state and miRNA expression**

A remarkable down-regulation of miR-99a and miR-205 was found in smoker patients (P= 0.04, P= 0.01, respectively) (Table 5). In addition, a direct and significant association was detected between smoking and muscle invasion (P= 0.03).

There was a significant association between the expression level of miR-99a in patients exposed to carcinogens such as asbestos and active chemical colors (P=0.04).

After a two-year period follow-up, recurrence was observed in 16 out of 36 patients (44.6%). No relationship was found between miR-99a and miR-205 expression and disease recurrence (P= 0.43, P= 0.29, respectively). There was a significant relationship between recurrence and MIBC (P= 0.01). Muscle invasion was observed in 10 out of 16 patients with disease recurrence (62.5%).

**Table 2:** Expression of miR-99a and miR-205 in tumor and healthy adjacent tissues

**Variables**	**Tumor Sample** **(delta Ct)**		**Healthy adjacent sample (delta Ct)**		
	Number	Mean ± SD¶	Number	Mean ± SD	P-value¶¶
**miR-99a**	36	12.05 ± 3.60	36	4.04 ± 2.33	<0.001
**miR-205**	36	4.05 ± 4.58	36	2.98 ± 2.23	0.38

¶ SD: Standard Deviation;

¶¶ Wilcoxon test

**Receiver operating characteristic (ROC) curve analysis for miR-99a and miR-205**

**Fig. 2:**
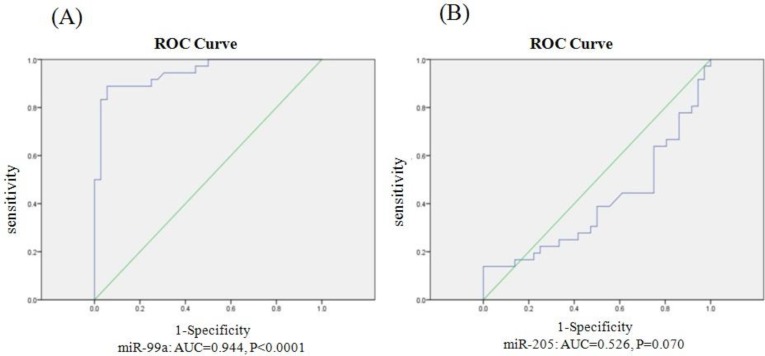
Receiver operating characteristic (ROC) curve analysis. A: miR-99a (AUC= 0.944, 95% confidence interval 0.87-1, specificity= 97%, sensitivity= 91%, P< 0.000); B: miR-205 (AUC= 0.526, P= 0.07). AUC: Area Under the Curve

ROC curve analysis was performed to determine the diagnostic value of miR-99a and miR-205 expression in discriminating tumor and healthy states of samples. Ideal cut off was identified by Youden index. ROC analysis for miR-99a showed area under the curve (AUC) value equal to 0.944 (95% CI 0.87-1, specificity of 97%, sensitivity of 91% and cut off value of 8.31), (P<0.001). ROC analysis for miR-205 was not significant (AUC= 0.526, P= 0.7) (Figure 2).

## Discussion

BC is the fourth and third most common cancer in developed countries and arnong Iranian men, respectively(1). The accurate mechanism of bladder tumorigenesis has not been clearly determined to date. To this end, there is an urgent need for understanding the process of genetic changes to identify diagnostic biomarkers and therapeutic targets (4, 12). Due to the unique characteristics of miRNAs, researchers have much attention to define miRNA profiles in tumor tissues (9, 12, 22). To the best of our knowledge, there are few studies similar to our study examining the expression level of miR-99a and miR-205 in BC, thus reporting their relationship with clinical and pathological features (21, 24). In patients suffering from cancer, the pathological features such as tumor invasion and grade are the important components in diagnosis, client-center therapy and follow-up (25, 26).

**Table 3:** Association between muscle invasion and miRNA expression levels

	**NMIBC** ¶¶ ** (delta Ct)**		**MIBC** **¶** ** (delta Ct)**		**Variable**
P- Value¶¶¶¶	Mean ± SD	Number	Mean ± SD¶¶¶	Number	
0.02	21.39 ±2.16	23	13.88±4.73	13	**miR-99a**
0.05	19.35±3.58	23	17 ±6.12	13	**miR-205**

¶¶ NMIBC: non muscle invasive bladder cancer;

¶¶¶ SD: Standard Deviation;

¶¶¶¶ Mann-Whitney test.

**Table 4:** Association between grade of the tumor and miRNA expression levels

**Variable**	**PUNLMP** ¶		**LG** ¶¶		**HG** ¶¶¶		
	Number	Mean (delta Ct)	Number	Mean (delta Ct)	Number	Mean (delta Ct)	P- Value¶¶¶¶
**miR-99a**	2	17.00	17	23.00	17	14.18	0.05
**miR-205**	2	15.75	17	19.32	17	18.00	0.87

¶ papillary urothelial neoplasm of low malignant potential;

¶¶ low grade;

¶¶¶ high grade;

¶¶¶¶ Kruskal-Wallis test.

**Table 5:** Association between smoking and the expression level of miRNAs.

**Variable**	**Smoker patients**		**Non- smoker patients**		**Fold change** **(Smoker/ Non smoker)**	
	Number	Mean (delta Ct)	Number	Mean (delta Ct)		P- Value¶
miR-99a	25	20.88	11	13.09	0.0004	0.04
miR-205	25	21.28	11	12.18	0.001	0.01

¶ Mann-Whitney test

According to our study, the overall miR-99a expression was significantly down-regulated in tumor samples compared to normal tissues. Nevertheless, there was no statistical significance about miR-205 expression. Our findings showed that miR-99a was underexpressed nearly 333-fold, presumably showing the strong role of miR-99a in BC suppression. This is consistent with the studies carried out by Chou et al. and Yougang Feng et al. (17, 20).

Several studies showed that miR-99a expression often decreases in most cases with prostate, bladder, uterus and lung cancers (18, 19, 27). Such a reduction was observed in almost all samples (35 out of 36) in our study.

Approximately 75% of bladder tumors were shown to be superficial at diagnosis times, meaning that the muscle is not involved, and the remaining 25 % invade the muscle (28). In the present study, 64 % and 36% of the patients were diagnosed as NMIBC and MIBC, respectively, which was similar to other studies. The miR-99a expression level in MIBC was higher than that in NMIBC, confirming the results of Wszolek’s investigation (29).

In comparison with low grade and papillary urothelial neoplasm of low malignant potential, miR-99a was overexpressed in high grade in our study, supporting its role in poor differentiation of tumor cells. In some cases, due to insufficient tissue specimens, misclassification of malignant samples was obtained by biopsy, in which the pathologist was unable to verify the correct conditions (22). Therefore, overexpression of miR-99a can be useful in assessing tumor invasion.

Considering the tumor suppressor role of miR-99a, its down regulation is expected with cancer progression. According to our results, miR-99a was found to be underexpressed in the early stage of BC, while increased miR-99a expression was detected in muscle invasive and high-grade tumors. This result can be justified by the presence of different targets for miR-99a at different stages of cancer. For example miR-99a plays a tumor suppressor role by targeting oncogenes such as *FGFR3*(18, 19), and consequently prevents cancer initiation. On the other hand, genes such as *ST5*, CTD small phosphatase-like gene, ras association domain-containing protein 1 tumor suppressors, which are down-regulated in invasive and metastatic malignancies, are the other targets of miR-99a, hence explaining the upregulation of miR-99a in MIBC and high grade tumors.

Our data indicated that the overall expression of miR-205 in tumor tissues was about 2 times lower than that in the normal tissue (58.4% of samples in details), but it was not statistically significant.

Despite 6.5-fold upregulation of miR-205 in MIBC in comparison with NMIBC, no significant relationship was found with muscle invasion. To our knowledge, no similar results were reported in this field.

In a study, Hezova et al. reported the tumor suppressor role of miR-205 in esophageal adenocarcinoma (30). In another study, it has been shown that miR-205 expression decreases in bladder cancer samples compared to normal samples (about 80% of the cases) (23). Another study indicated the increased expression of miR-205 in BC tissue compared with the healthy tissue (22).

Different results of the miR-205 expression is likely due to different circumstances and in particular, factors affecting epigenetic changes in the study population (30) such as different geographic regions, lifestyle, as well as different study design, sample size and tumor grade and stage, which can affect the results of studies based on human participants (30).

In a meta-analysis study, the dual role of miR-205 in different cancers has been reported. The majority of studies indicated the tumor suppressor activity of miR-205 in different types of cancer. At the same time, deregulation of miR-205 can cause another tumor (30). The lack of significant expression of miR-205 in this study and controversial results in similar studies may show a complex impression of miR-205 in cancer initiation and progression.

Despite the different findings of miR-205, specific genes such as *ZEB1*, *ZEB2*, and E-Cadherin which are targeted by this miRNA, play an important role in the formation and progression of BC (15, 16).

Smoking is one of the most well-known causes of bladder cancer (31). Because of decreased expression of miR-99a and miR-205 in BC tissue samples and underexpression of these miRNAs in smoker patients, it might be concluded that smoking is one of the important factors in BC carcinogenesis through epigenetic alterations.

Cystoscopy is the most reliable method of diagnosis of bladder cancer at present, which is a costly, invasive and time-consuming procedure. Yet CT scans and MRI are normally used for tumor staging in muscle invasive tumors, these imaging methods in some cases, for example, the separation of the bladder tumor at stage T2 and T3a are confronted with an error (32, 33). Transurethral resection of bladder tumor accompanied by histological assessment are still the main method for the diagnosis of invasive bladder cancer, particularly invasive bladder cancer in the early stages (12, 34).

According to ROC curve analysis for miR-99a, we obtained an AUC equal to 0.944 which is a high rate for a diagnostic test. As a consequence, miR-99a can be used as a reliable diagnostic biomarker for BC.

Due to the foregoing points, normal expression of miR-99a is supposed to have tumor suppressor ability. MiR-99a was also associated with bladder tumor invasion. As a result, it can be suggested as a biomarker for diagnostic approach and targeting therapy as well as to determine the progression state.

Certainly, it is necessary to conduct further studies on miRNA alterations by simultaneously evaluating the expression of their targets with a larger sample size and appropriate number of all stages and grades to clarify the impact of these two miRNAs in all processes of bladder carcinogenesis.

In this case- control study we assessed BC and adjacent normal tissue without considering age, sex and other demographic data. Besides, we matched and compared each cancer and normal tissue from the same participant; accordingly, age and gender were not confounding bias factors. Though, most cases were males (33 males vs 3 females) then we could not evaluate the association between micro-RNA and sex and age.

In conclusion**, **dramatic down-regulation of miR-99a in bladder cancer tissues confirms the tumor suppressor role of miR-99a in the bladder cancer. The higher amount of miR-99a expression was associated with invasive bladder cancer. Based on ROC analysis, miR-99a could be suggested as a diagnostic biomarker. The lack of significant expression of miR-205 in this study and controversial results in similar studies may show the complex role of miR-205 in bladder cancer.
